# Supplementary material for: DMRforPairs: identifying Differentially Methylated Regions between unique samples using array based methylation profiles
Source: BMC Bioinformatics. 2014 May 15;15:141. doi: 10.1186/1471-2105-15-141 (PMC4046028; doi:10.1186/1471-2105-15-141)
Supplement: Additional file 2 — DMRforPairs output for the comparison of A431-MCF7 and NA17018-NA17105. Please start from the HTML files in each folder. Available via the BMC Bioinformatics website. [file 1471-2105-15-141-S2.zip › 1394847754114233_MOESM2_ESM/A431_MCF7/figures/5422.pdf]

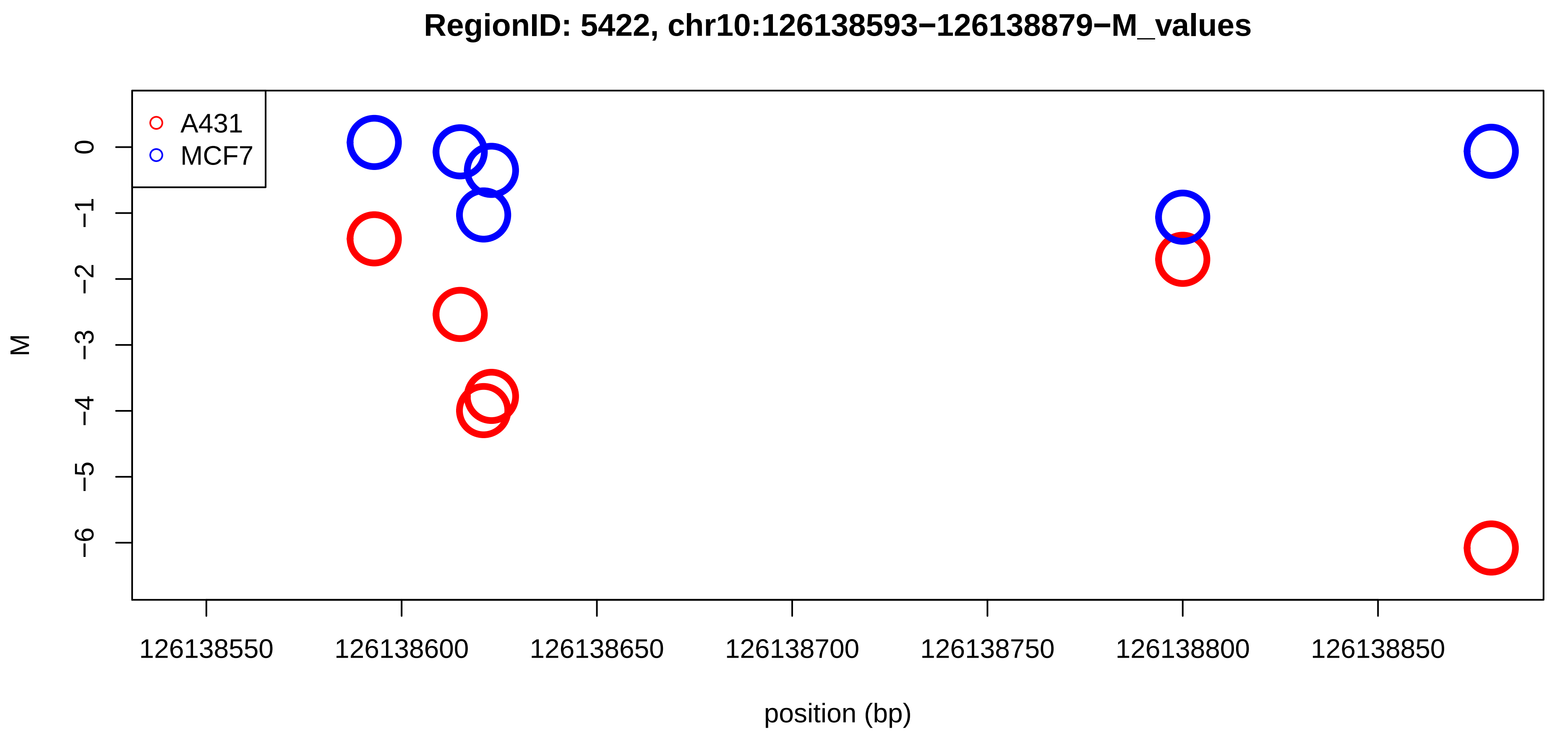
RegionID: 5422, chr10:126138593−126138879−M_values
A431
MCF7
M
0
−1
−2
−3
−4
−5
−6
126138550
126138600
126138650
126138700
126138750
126138800
126138850
position (bp)

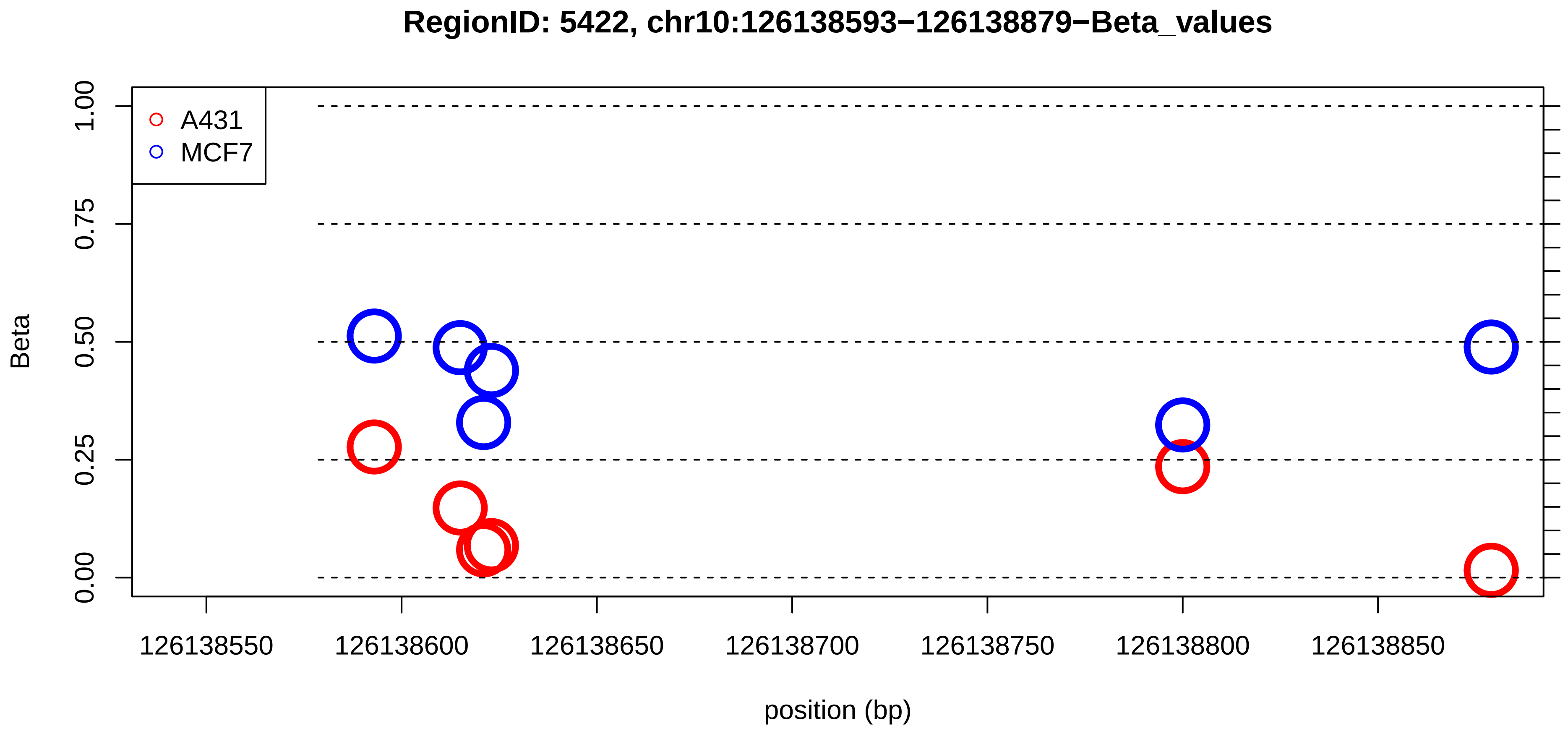
RegionID: 5422, chr10:126138593−126138879−Beta_values
A431
MCF7
Beta
0.00
0.25
0.50
0.75
1.00
126138550
126138600
126138650
126138700
126138750
126138800
126138850
position (bp)

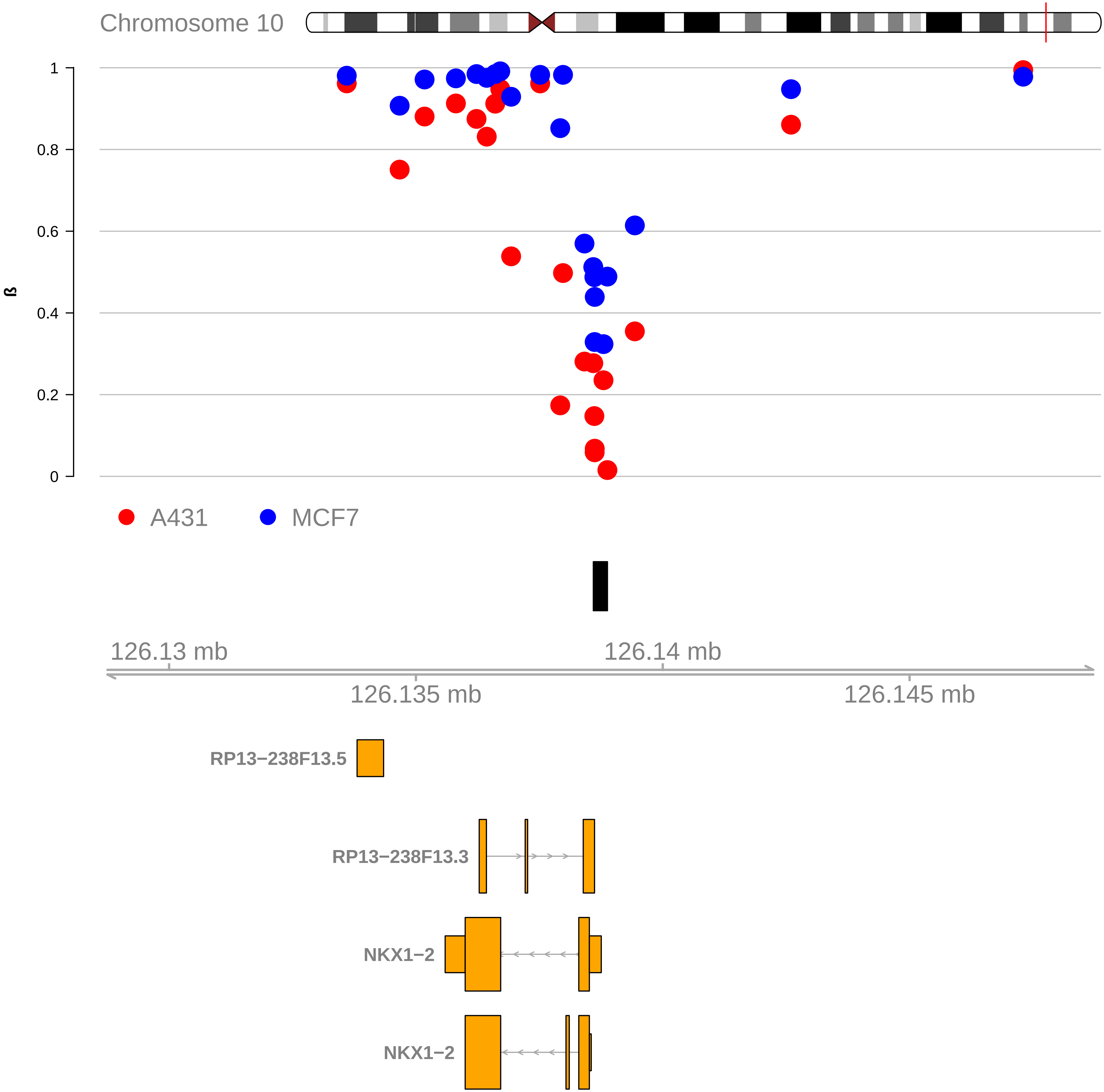

Chromosome 10
ß
1
0.8
0.6
0.4
0.2
0
A431
MCF7
126.13 mb
126.14 mb
126.135 mb
126.145 mb
RP13−238F13.5
RP13−238F13.3
NKX1−2
NKX1−2
